# Supplementary material for: Effectiveness of Different Types of Core Decompression in Early-Stage Osteonecrosis of the Femoral Head: A Systematic Review and Meta-Analysis
Source: Med Sci (Basel). 2025 Nov 3;13(4):258. doi: 10.3390/medsci13040258 (PMC12641739; doi:10.3390/medsci13040258)
Supplement: Supplementary file 1 [file medsci-13-00258-s001.zip › medsci-3946186-supplementary.pdf]

Supplementary Materials

Search strategy

PubMed

("Avascular Necrosis of Femoral Head"[Mesh] OR "Osteonecrosis of the Femoral Head" OR "Femoral Head Necrosis" OR AVNFH OR ONFH) AND ("Core Decompression"[Mesh] OR "Core Decompression" OR "Decompression Surgery")

EMBASE

('avascular necrosis of bone'/exp OR 'avascular necrosis':ti,ab OR 'osteonecrosis':ti,ab

OR 'femoral head necrosis':ti,ab OR 'avnfh':ti,ab OR 'onfh':ti,ab

OR 'avascular necrosis of femoral head':ti,ab)

AND

('femoral head'/exp OR 'femoral head':ti,ab OR 'hip joint'/exp OR hip:ti,ab)

AND

('core decompression'/exp OR 'core decompression':ti,ab OR decompression:ti,ab

OR drilling:ti,ab OR 'bone drilling':ti,ab)

AND

(stage:ti,ab OR early:ti,ab OR 'ficat':ti,ab OR 'arco':ti,ab)

NOT ('animal'/exp NOT 'human'/exp)

NOT ('systematic review'/exp OR 'review'/exp)

AND [article]/lim

AND [humans]/lim

AND [adult]/lim

AND [2000-2025]/py

Web of Science

TS=(

(("avascular necrosis" OR osteonecros* OR "femoral head necrosis" OR AVNFH OR ONFH) NEAR/3 ("femoral head" OR hip))

AND ("core decompression" OR (drill* NEAR/3 decompression) OR "multiple drilling")

AND (early OR "early-stage" OR "pre-collapse" OR precollapse OR "Ficat I" OR "Ficat II" OR "ARCO I" OR "ARCO II")

)

Cochrane library

("core decompression" OR "core decompression surgery") AND

("avascular necrosis of femoral head" OR "AVNFH" OR "osteonecrosis of femoral head" OR "ONFH") AND

("early stage" OR "pre-collapse")
